# Sustained Spindle-Assembly Checkpoint Response Requires *De Novo* Transcription and Translation of Cyclin B1

**DOI:** 10.1371/journal.pone.0013037

**Published:** 2010-09-28

**Authors:** Ana Lúcia Mena, Eric W.-F. Lam, Sukalyan Chatterjee

**Affiliations:** 1 Instituto Gulbenkian de Ciência, Oeiras, Portugal; 2 Department of Cancer and Surgery, Imperial College London, London, United Kingdom; University of Minnesota, United States of America

## Abstract

**Background:**

Microtubule-targeting drugs induce mitotic delay at pro-metaphase by preventing the spindle assembly checkpoint to be satisfied. However, especially after prolonged treatments, cells can escape this arrest in a process called mitotic slippage. The mechanisms underlying the spindle assembly checkpoint and slippage are not fully understood. It has been generally accepted that during mitosis there is a temporary shutdown of high-energy-consuming processes, such as transcription and translation. However, the synthesis of specific proteins is maintained or up-regulated since protein synthesis is necessary for entry into and progression through mitosis.

**Methodology/Principal Findings:**

In this work we investigated whether the mitotic arrest caused by the mitotic checkpoint is independent of transcription and translation. By using immunofluorescent microscopy and western blotting, we demonstrate that inhibition of either of these processes induces a shortening of the mitotic arrest caused by the nocodazole treatment, and ultimately leads to mitotic slippage. Our western blotting and RTQ-PCR results show that inhibition of transcription during mitotic arrest does not affect the expression of the spindle checkpoint proteins, whereas it induces a significant decrease in the mRNA and protein levels of Cyclin B1. The exogenous expression of Cyclin B1 substantially rescued the mitotic phenotype in nocodazole cells treated with the inhibitors of transcription and translation.

**Conclusions/Significance:**

This work emphasizes the importance of transcription and translation for the maintenance of the spindle assembly checkpoint, suggesting the existence of a mechanism dependent on *cyclin B1* gene regulation during mitosis. We propose that continuous transcription of mitotic regulators is required to sustain the activation of the spindle assembly checkpoint.

## Introduction

The spindle assembly checkpoint (SAC), or mitotic checkpoint, monitors the microtubule attachment to kinetochores, ensuring the correct segregation of chromosomes [1], [2]. Therefore, this mechanism prevents sister chromatids separation, delaying the onset of anaphase, until all kinetochores are correctly attached to the mitotic spindle and under appropriate tension. Unattached kinetochores trigger a diffusible signal [2], [3], the mitotic checkpoint complex (MCC), that inhibits the Anaphase Promoting Complex/Cyclosome (APC/C) by acting as a pseudo-substrate of APC/C (reviewed in ref. [4]). Besides Mad2, BubR1, Bub3 and Cdc20, that compose the MCC [5], several other proteins, including other SAC proteins (e.g. Mad1, Mps1 and Bub1) play an important role in the recruitment and establishment of the MCC (reviewed in ref. [4]). Once the proper attachment of all kinetochores to the mitotic spindle occurs, the SAC becomes satisfied and is inactivated. The Cdc20 activates the APC/C which promotes the ubiquitination of several mitotic substrates, including Cyclin B1 and Securin, that will be subsequently degraded by the proteasome (reviewed in ref. [6]).

Spindle poisons disturb the dynamics of microtubules (MTs), either by depolymerisation (e.g. nocodazole) or by stabilization (e.g. taxol; at high concentration induces polymerization) [7], [8], resulting in lack of attachment from the kinetochores. Consequently, the SAC remains activated and cells are arrested in pro-metaphase. The duration of mitotic delay depends on several factors, including the organism, the cell type and the initial concentration of the spindle poison (reviewed in ref. [9]). However, especially after prolonged treatments, cells may overcome the mitotic delay, even in the presence of the drug, and cycle into G1 without achieving cytokinesis, in a process named mitotic slippage [8], [9], [10], [11], [12]. Therefore, the nuclear envelope reforms around random groups of chromosomes as the chromosomes decondense and the adapted cells become multinucleated and tetraploid. Even though the molecular details that underlie mitotic slippage are not fully understood, Cyclin B degradation and the consequent inactivation of the cyclin-dependent kinase 1 (CDK1) has been shown to play an important role [13].

During mitosis, there is a temporary shutdown of high-energy-consuming processes, such as transcription and translation. The concept of a lack of transcription in mammalian cells during mitosis has been generally accepted for more than 40 years (reviewed in ref. [14]). Similarly, the rate of protein synthesis decreases in mitosis. However, the synthesis of specific proteins are maintained or up-regulated since protein synthesis is necessary for entry into and progression through mitosis (reviewed in refs. [15], [16]). Contrary to the widely accepted notion that transcription is repressed during mitosis in higher eukaryotes, the active transcription of *cyclin B1* gene during mitosis has been proposed in HeLa cells [17]. Here, we investigated whether SAC is independent of *de novo* transcription and translation processes. Our results indicate that both transcription and translation of Cyclin B1 are required for the sustained activation of the SAC.

## Results

### Inhibition of transcription and translation induces mitotic slippage

**Figure 1:**
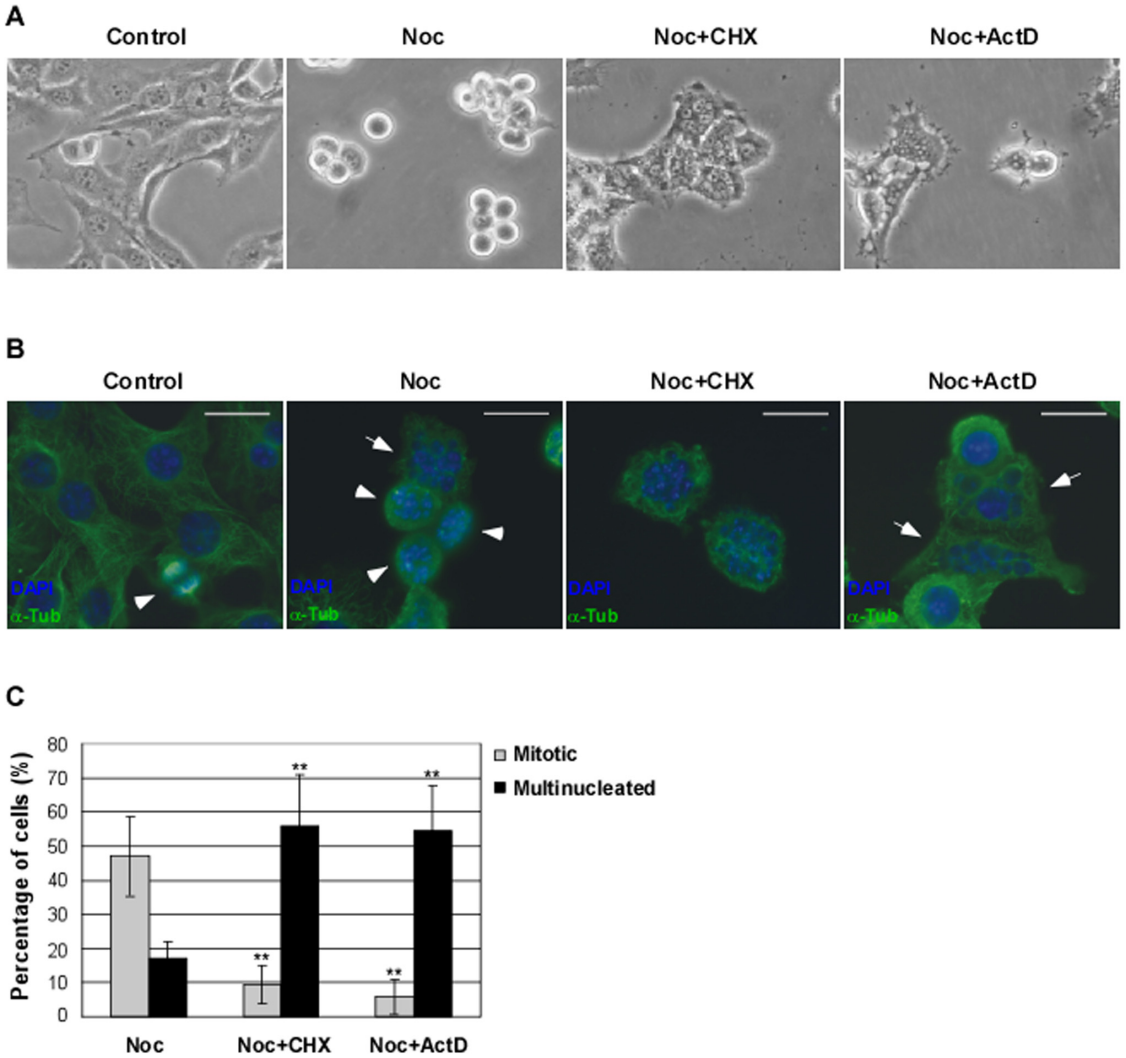
Mitotic slippage is induced in nocodazole-treated cells after inhibition of transcription or translation. Characterization of NIH3T3 cells: control cells, nocodazole (noc)-treated cells (arrested in mitosis), noc-cells incubated with cycloheximide (CHX; 35.5 µM) or actinomycin D (ActD; 8 µM) for 4 h. A) Phase-contrast microscopy; magnification: 200×. B) Immunofluorescence microscopy; arrowheads indicate mitotic cells and arrows indicate cells that underwent mitotic slippage; DAPI stains the DNA (blue) and anti-α-tubulin antibody stains the microtubules (green). Scale bar  = 25 µm. C) Mitotic and multinucleation indexes of noc-treated cells with or without CHX or ActD for 4 h. Data presented as mean ± S.D. from three independent experiments; ** p<0.01 noc-CHX or noc-ActD *versus* noc.

**Figure 2:**
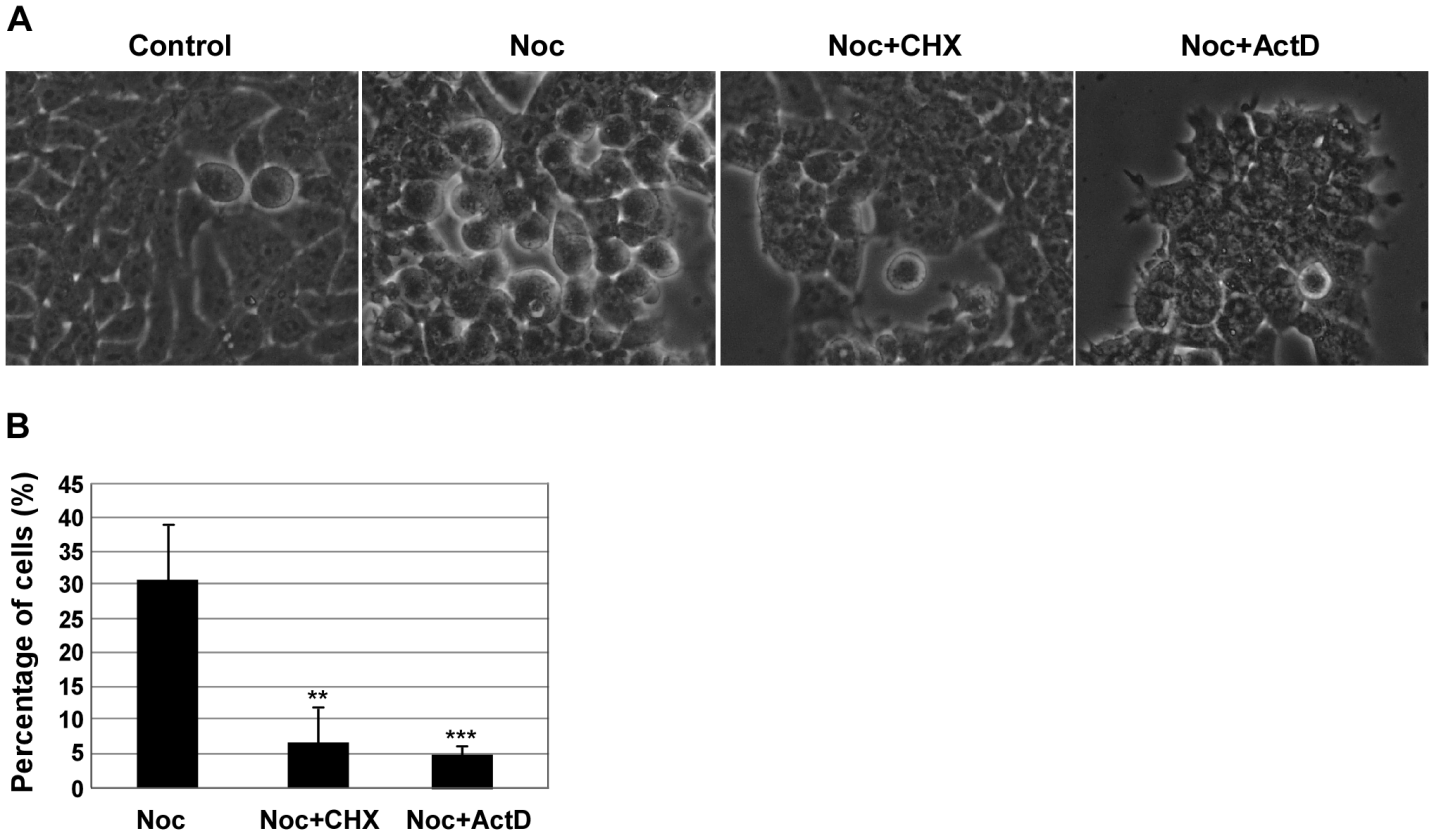
Inhibition of transcription or translation induces mitotic slippage in nocodazole treated HEK293 cells. Characterization of HEK293 cells: control cells, nocodazole (noc)-treated cells (arrested in mitosis), noc-cells incubated with cycloheximide (CHX; 35.5 µM) or actinomycin D (ActD; 8 µM) for 6 h. A) Phase-contrast microscopy; magnification: 200×. B) Mitotic index of noc-treated cells with or without CHX or ActD for 6 h. Data presented as mean ± S.D. from three independent experiments; ** p<0.01 and *** p<0.005 noc-CHX or noc-ActD *versus* noc.

In the presence of an active SAC, treatment with drugs that disturb microtubule assembly, such as nocodazole, causes cell-cycle arrest in pro-metaphase [7], but the molecular details behind such event are not completely clarified. We investigated whether the mitotic delay caused by treatment with spindle poisons could occur independently of transcription and translation. NIH3T3 cells were incubated for 14 h with nocodazole and further treated for 4 h with either 35.5 µM of cycloheximide (CHX), a *de novo* protein synthesis inhibitor [18], or 8 µM of actinomycin D (ActD), an inhibitor of transcription [19]. Morphological visualization by phase-contrast microscopy showed that, in comparison to nocodazole-arrested cells (noc-cells; incubated with nocodazole for 18 h), both CHX treated noc-cells (noc-CHX cells) and ActD treated noc-cells (noc-ActD cells) lost the rounded morphology characteristic of mitotic cells and became flat and adherent (Fig. 1A). Furthermore, microtubule staining showed loss of disassembled mitotic spindle (characteristic of nocodazole treatment) and DNA staining with DAPI revealed the presence of multinuclei and/or micronuclei (Fig. 1B; arrowheads indicate mitotic cells whereas arrows show multinucleated cells). This morphology is in agreement with previous description of cells that slipped through mitosis, namely after a prolonged mitotic delay in the presence of spindle poisons [11], [12], [20]. Calculation of the mitotic index by analysis of condensed chromosomes showed that noc-cells presented a index of approximately 50% whereas after treatment with CHX or ActD the mitotic index decreased to values of approximately 10%, corresponding to a fold decrease of approximately 80% (p<0.01, noc-CHX and noc-ActD cells compared to noc-cells) (Fig. 1C). Both the CHX and ActD treatments induced almost 40% increase in the number of multinucleated cells in the total cell population. Hence, we observed that the length of the SAC was reduced, increasing the number of cells that escape mitosis (Fig. 1). (Note that a small number of noc-cells also slipped after the 18 h treatment with nocodazole [Fig. 1B and C]). The effect of inhibition of transcription and translation in the SAC length was also addressed in the human cell line HEK293 (Fig. 2). HEK293 cells were incubated for 14 h with nocodazole and further treated for 6 h with either 35.5 µM of CHX or 8 µM of ActD. Again, calculation of the mitotic index revealed a statistically significant decrease in noc-CHX and noc-ActD cells compared to noc-cells (p<0.01 and p<0.005 respectively) (Fig. 2B).

These results suggest that transcription and translation are required to maintain cells arrested in mitosis and that pharmacological inhibition of either process can reduce the length of the SAC inducing mitotic slippage.

### Inhibition of transcription and translation results in the activation of the APC/C

Inactivation of the SAC normally leads to the activation of the APC/C^Cdc20^ (reviewed in ref. [4]). In order to address whether the APC/C^Cdc20^ was activated during the slippage caused by inhibition of transcription or translation we blocked 26S proteasome-dependent degradation using the pharmacological inhibitor MG132. As previously demonstrated in other systems [13], [21], exposure to MG132 prevented mitotic slippage induced by CHX and ActD (data not shown).

**Figure 3:**
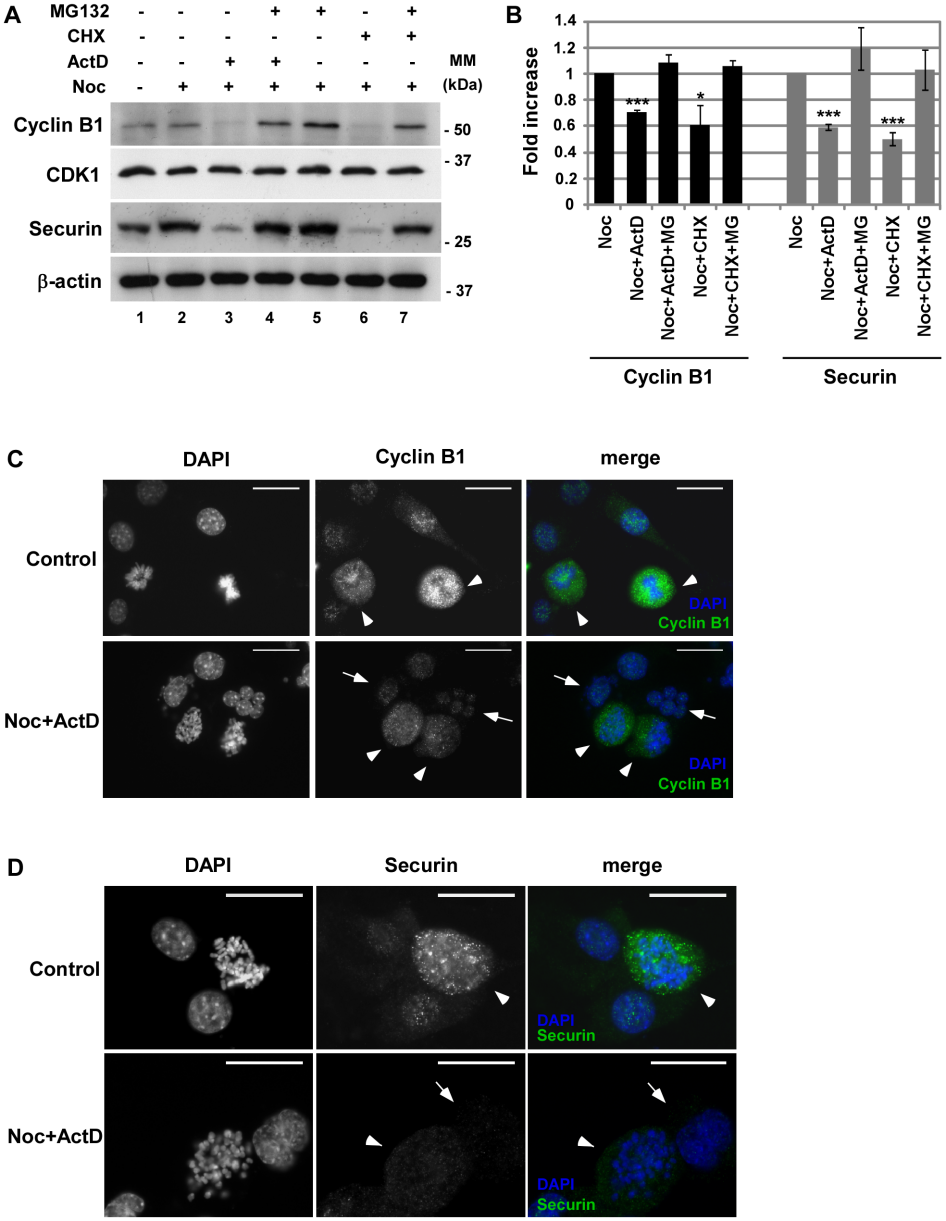
Cyclin B1 and Securin are degraded during mitotic slippage induced by inhibition of transcription and translation. A) Analysis of Cyclin B1, Securin and CDK1 levels by western blotting. NIH3T3 cells were incubated with nocodazole (noc) for 14 h and treated with actinomycin D (ActD; 8 µM) or cycloheximide (CHX; 35.5 µM) with or without the inhibitor of the proteasome MG132 (25 µM), for 4 h; β-actin was used as loading control. B) Quantification of Cyclin B1 and Securin proteins in noc-, noc-ActD-, noc-ActD-MG, noc-CHX and noc-CHX-MG-cells. Normalization of Cyclin B1 and Securin was calculated on the ratio of these proteins per β-actin protein present in each condition. Data presented as mean ± S.D. from three independent experiments. C and D) Immunofluorescence microscopy: asynchronous population (control) and noc-cells treated with ActD for 4 h; DAPI stains the DNA (blue) and Cyclin B1 (C) and Securin (D) are stained in green. Arrowheads point mitotic cells and arrows point cell that undergone mitotic slippage. The adapted cells shown are representative of both ActD and CHX induced mitotic slippage. Scale bar  = 25 µm.

Next, we investigated if two substrates of APC/C^Cdc20^, Cyclin B1 and Securin, were being degraded. Treatment of noc-cells with ActD or CHX for 4 h resulted in the decrease of both Cyclin B1 and Securin at protein levels, revealed by western blotting (Fig. 3A, lane 3 and 6 compared to lane 2). Figure 3B shows the quantification of Cyclin B1 and Securin protein levels. Staining of Cyclin B1 and Securin by immuno-fluorescence microscopy (Fig. 3C and D) confirmed that cells that slipped mitosis (indicated by arrows) contain lower amounts of Cyclin B1 and Securin than mitotic cells (indicated by arrowheads). Incubation with the proteasome inhibitor MG132 restored the levels of Cyclin B1 and Securin (Fig. 3A, lanes 4 and 7). We next analysed the levels of CDK1 that have been shown to decrease in other systems of mitotic slippage [11]. However, the levels of CDK1 remained unaffected, despite the absence of transcription or translation during mitotic delay (Fig. 3A). Together, our data suggests that inhibition of transcription and translation during SAC arrest induced by nocodazole results in the activation of the APC/C^Cdc20^ and the subsequent degradation of Securin and Cyclin B1. These results are in concordance with recent observations of activation of APC/C during mitotic slippage [22].

### Inhibition of transcription affects *cyclin B1* gene expression

**Figure 4:**
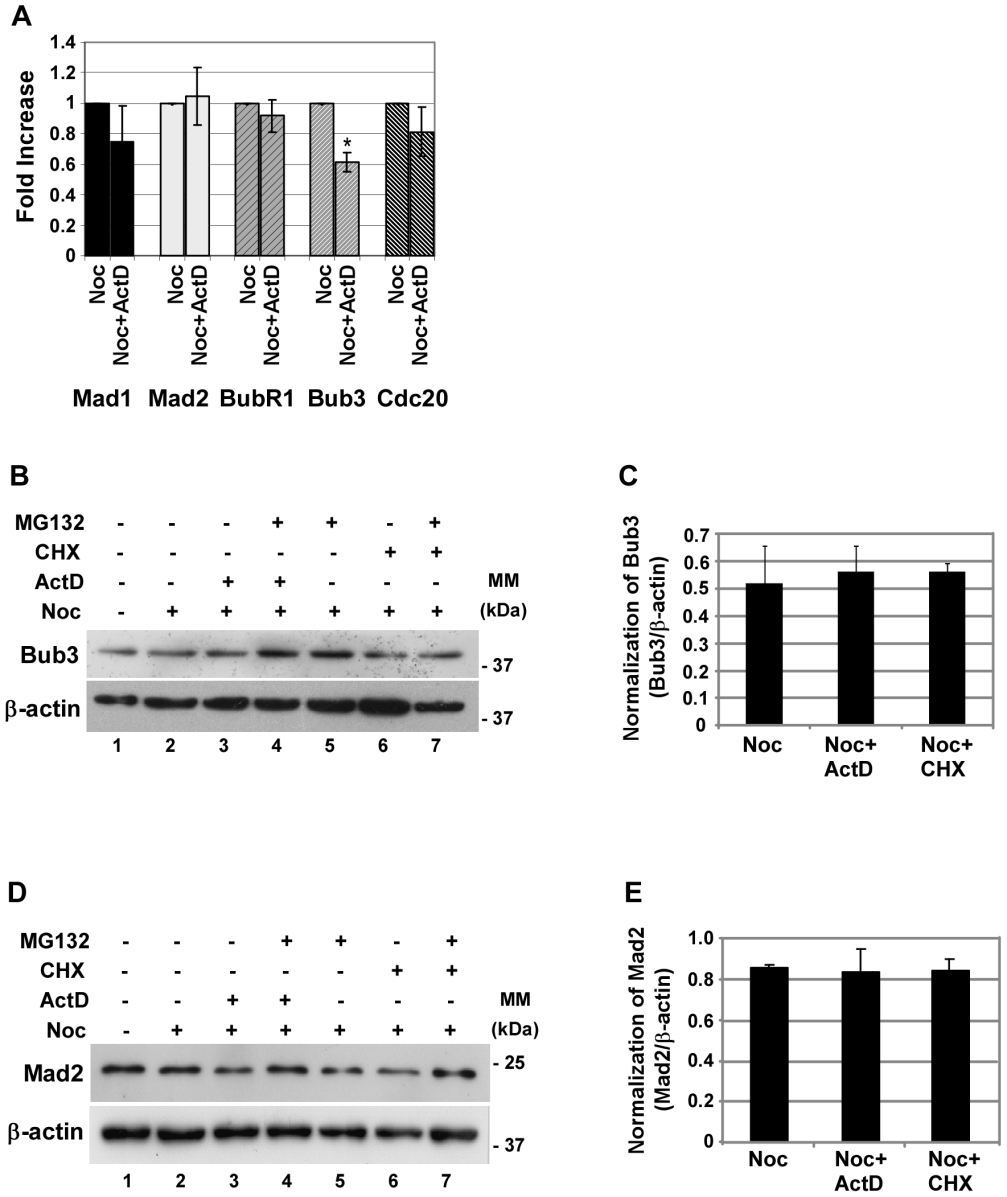
Expression of Mad1, Mad2, BubR1 and Cdc20 are not affected by inhibition of transcription, whereas Bub3 transcript levels are affected but Bub3 protein is not. A) Real time quantitative PCR analysis of several spindle checkpoint transcripts (Mad1, Mad2, BubR1, Bub3 and Cdc20) present in nocodazole (noc) cells treated with or without the inhibitor of transcription actinomycin D (ActD; 8 µM), for 4 h. Results from each gene amplification were normalized using L19 expression and presented as fold increase from nocodazole cells. Data presented as mean ± S.D. from three independent experiments; * p<0.05 noc-ActD *versus* noc. B and D) Western blotting analysis of Bub3 (B) and Mad2 (D) in nocodazole (noc) cells treated with ActD or cycloheximide (CHX; 35.5 µM) in the presence or absence of the proteasome inhibitor MG132 for 4 h; β-actin was used as loading control. C and E) Quantification of Bub3 (C) and Mad2 (E) protein in noc-, noc-ActD- and noc-CHX-cells. Normalization of Bub3 or Mad2 was calculated on the ratio of these proteins per β-actin protein present in each condition. Data presented as mean ± S.D. from three independent experiments.

Since it has been described previously that translation of specific proteins is maintained or up-regulated during mitosis [15], we hypothesized that both transcription and translation of at least one protein responsible for the mitotic delay would occur during mitosis. Previously, it has been shown that cells depleted of Mad2, Bub3, Rae1, BubR1 or CENP-E are unable to sustain a pro-metaphase arrest in the presence of spindle damage [23], [24]. Furthermore, deregulation of spindle checkpoint proteins can induce premature mitotic exit, even in the presence of spindle drugs [20], [25], [26]. In order to address if inhibition of transcription would affect the expression of spindle checkpoint transcripts, *bubR1*, *bub3*, *mad1*, *mad2* and *cdc20* gene expression were analysed by real-time quantitative PCR. The result showed that Bub3 mRNA levels significantly decreased (p<0.05) in the presence of ActD, whereas the levels of Cdc20, Mad1, Mad2 and BubR1 transcripts did not display significant differences (Fig. 4A). Reduction in Bub3 mRNA has been reported in cells upon prolonged exposure to taxol, and been proposed as a cause to the mitotic slippage [11]. As a result, we next investigated if the decrease of Bub3 transcripts was associated with a parallel decrease in the protein levels, which can lead to the activation of the APC/C^Cdc20^. Contrary to what happens at the mRNA level, western blotting analysis showed no relevant change in the protein levels of Bub3 from noc-CHX or noc-ActD cells (Fig. 4B and C, lanes 3 and 6 compared to 2). The reason for this is unclear; however, it is possible that the Bub3 protein pool has a low turnover and, therefore, is not affected by the inhibition of *bub3* transcription.

To confirm that the negative effect of inhibition of transcription on the expression of SAC transcripts analysed was also reflected at the protein level, we analysed the protein levels of Mad2. Consistent with the lack of variation observed at the transcriptional level, inhibition of transcription and translation did not significantly affect the protein levels of Mad 2 (Fig. 4D and E, lanes 3 and 6 compared to lane 2).

**Figure 5:**
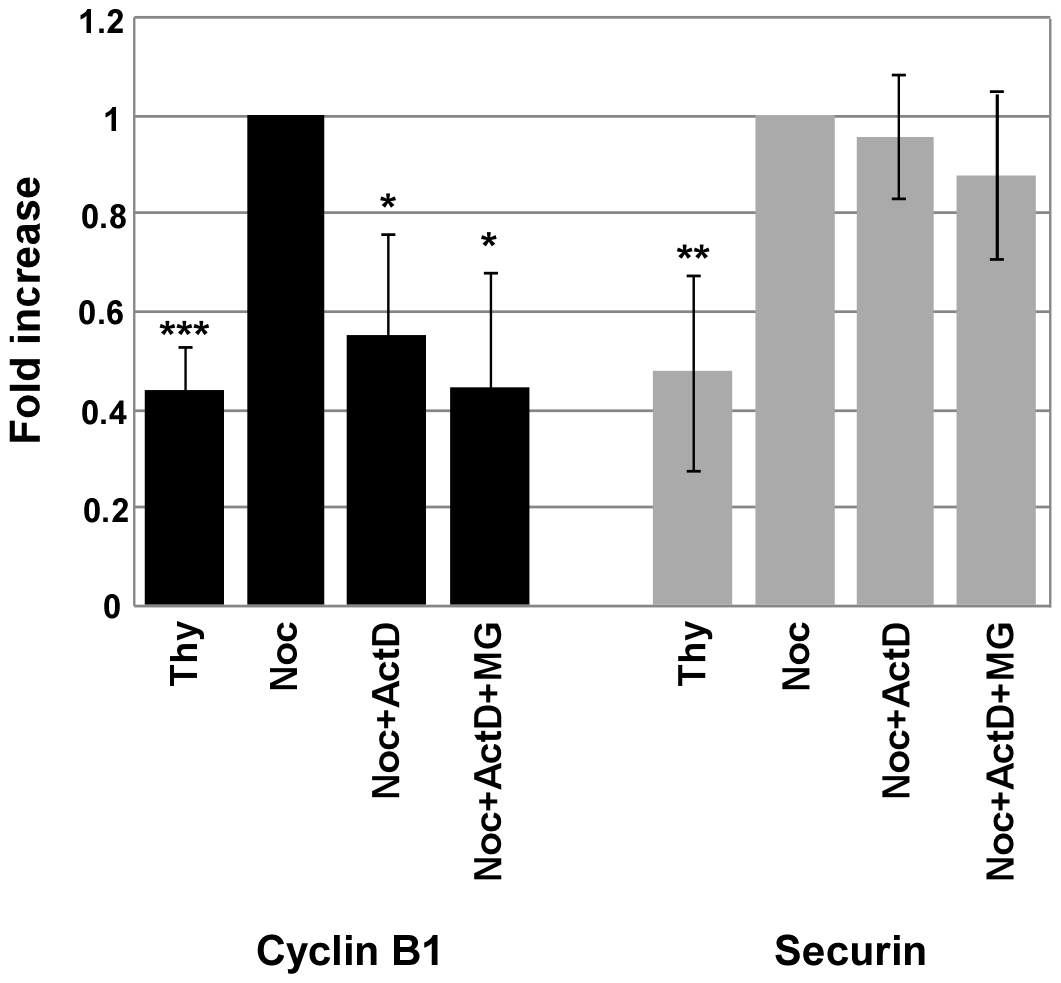
Inhibition of transcription results in the decrease of Cyclin B1 mRNA levels, but do not affect Securin's transcripts. Analysis of the level of transcripts of Cyclin B1 and Securin by real-time quantitative PCR. mRNA was isolated from a G1/S enriched population of cells treated with thymidine (Thy), and from nocodazole- arrested cells (noc-cells) treated with or without actinomycin D (ActD; 8 µM) and MG132 for 4 h. Results from Cyclin B1 and Securin amplification were normalized using L19 expression and presented as fold increase of Cyclin B1 or Securin expression from noc-cells. Data presented as mean ± S.D. from three independent experiments; *** p<0.005; ** p<0.01; * p<0.05 Thy or Noc+ActD or Noc+ActD+MG *versus* Noc.

Given that Cyclin B1 and Securin protein levels were down-regulated, we next investigated if inhibition of transcription was affecting the expression of *cyclin B1* and *Pttg* (the gene that codes for Securin) genes. To this end, mRNA was isolated from a population of cells synchronized at the G1/S boundary with thymidine (Thy), noc-cells, noc-ActD cells and noc-ActD-MG cells, and the levels of Cyclin B1 and Securin transcripts analysed by real-time quantitative PCR. The result showed a significant decline (p<0.05) in the mRNA levels of Cyclin B1 when transcription was inhibited during the mitotic delay, whereas the levels of Securin transcripts did not show significant differences (Fig. 5).

**Figure 6:**
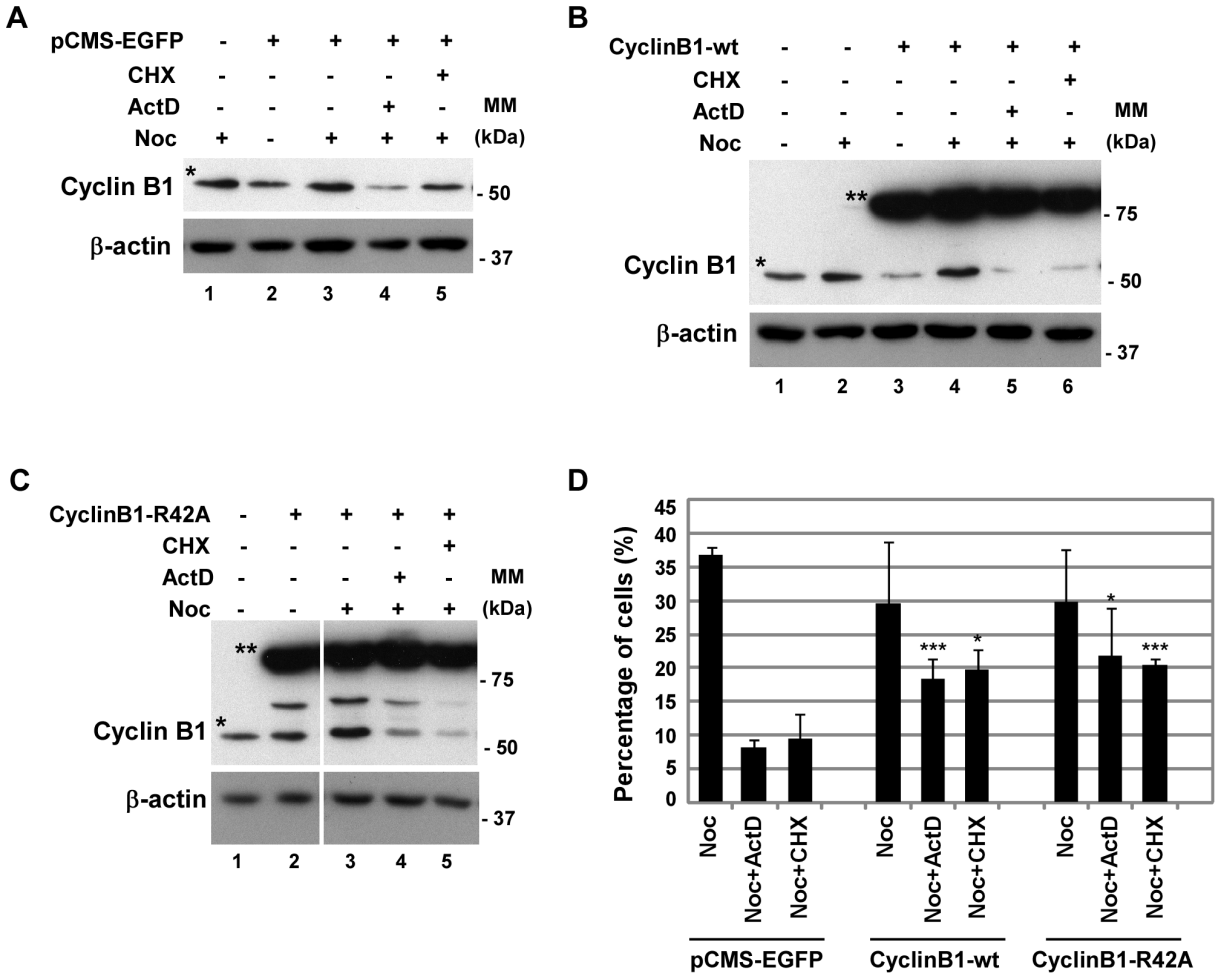
Overexpression of Cyclin B1 protein rescues the mitotic phenotype. HEK293 cells were transfected with the wild-type and the non-degradable forms of Cyclin B1 (CyclinB1-wt and CyclinB1-R42A, respectively); pCMS-EGFP was used as transfection control. A, B and C) Analysis of Cyclin B1 endogenous (indicated with one asterisk) and exogenous (indicated with double asterisk) protein levels by western blotting in cells transfected with pCMS-EGFP (A), CyclinB1-wt (B) and CyclinB1-R42A (C). After transfection, HEK293 cells were incubated with nocodazole (noc) for 14 h and treated with actinomycin D (ActD; 8 µM) or cycloheximide (CHX; 35.5 µM) for 6 h; β-actin was used as loading control. All lanes presented in C are from the same experiment. D) Mitotic index of nocodazole (Noc) treated cells with or without CHX or ActD for 6 h. Data presented as mean ± S.D. from three independent experiments; *** p<0.005 and *p<0.05 noc-CHX or noc-ActD from cells transfected with Cyclin B1 *versus* noc-CHX or noc-ActD from cells transfected with pCMS-EGFP.

To further investigate the role of Cyclin B1 expression in the maintenance of the SAC activation we attempted to rescue the mitotic phenotype by transient transfection of HEK293 cells with wild type and non-degradable forms of Cyclin B1 (CyclinB1-wt and CyclinB1-R42A, respectively), using as control pCMS-EGFP (Fig. 6). The transfection efficiency was approximately 50%, and all subsequent analyses were performed in the mixed population of transfected and non-transfected cells. Western blotting analysis revealed that treatment of noc-cells with ActD or CHX for 6 h resulted in the decrease of endogenous Cyclin B1 protein levels but did not affect the exogenous expression of Cyclin B1 (Fig. 6B and C; lanes 5 and 6 compared to lane 4 in panel B, lanes 4 and 5 compared to lane 3 in panels C; endogenous Cyclin B1 indicated with an asterisk whereas exogenous Cyclin B1 is indicated with a double asterisk). Moreover, the decrease of endogenous Cyclin B1 protein in CyclinB1 transfected cells was similar to the one observed in pCMS-EGFP transfected cells. Calculation of the mitotic index revealed that, upon treatment with ActD or CHX, cells transfected with either CyclinB1-wt or CyclinB1-R42A approximately doubled the mitotic figures present in the cells transfected with pCMS-EGFP (p<0.05 and p<0.005) (Fig. 6D), rescuing the mitotic phenotype. Notably, the standard deviation observed may result from differences in the transfection efficiency. Also, the transfection efficiency of 50% may be preventing a more substantial rescue of the mitotic phenotype. This result indicates that if Cyclin B1 protein levels are increased within the cell, during the same period of time, there are less cells slipping mitosis.

Overall our data suggest that inhibition of transcription and translation induces the inactivation of SAC by affecting the expression of Cyclin B1.

## Discussion

Here, we show that both transcription and translation are required for the maintenance of the mitotic delay. We show that four and six hours of treatment with actinomycin D or cycloheximide is sufficient to override the mitotic delay caused by nocodazole in NIH3T3 and HEK293 cells, respectively, and induce slippage. The mechanism underlying slippage in our system seems to be independent of changes in the expression of Mad1, Mad2, BubR1, Cdc20, Securin and Bub3, although Bub3 mRNA levels were affected upon inhibition of transcription but no significant change was observed at the protein level. Our results suggest that the mechanism underlying the activation of the APC/C^Cdc20^ upon mitotic inhibition of transcription or translation involves *cyclin B1* gene expression.

Our work questions the prevailing model of the general inhibition of transcription during mitosis (reviewed in ref.[14]), since we clearly show that pharmacological inhibition of transcription during mitotic delay results in the abrogation of the mitotic delay. However, our work is in accordance with the observation that transcription of *cyclin B1* is active during mitosis [17]. The decrease of Cyclin B1 mRNA levels upon inhibition of transcription during mitotic delay and the mitotic phenotype rescue by overexpression of Cyclin B1 protein indicate that Cyclin B1 expression during mitosis indeed is crucial for the sustained activation of the SAC. Although we cannot exclude the fact that the spindle checkpoint proteins might have a role in the mechanism underlying the mitotic slippage induced by inhibition of transcription or translation through relocation, our results do indicate that this mechanism is independent of changes in the expression of the spindle checkpoint proteins analysed.

The proposal of the requirement of Cyclin B1 expression during mitosis for the sustained activation of the SAC fits within the continuous Cyclin B1 degradation model [13]. This model has been proposed based on the observation of a slow but continuous proteasome-dependent proteolysis of Cyclin B, in the presence of an active SAC. In the presence of microtubule targeting drugs SAC cannot be satisfied and bypassing an active SAC requires proteasome-mediated proteolysis of Cyclin B1 to escape mitosis. The continuous degradation model proposes that, in vertebrate cells, the SAC would not efficiently block all APC/Cs from ubiquitinating Cyclin B, leading to a gradual drop of Cyclin B until it reaches a level lower than the needed to maintain the mitotic delay [13]. We extend this model and propose that the decrease in the Cyclin B1 mRNA levels observed in our data by inhibition of transcription may contribute to a further decrease in the levels of the protein pool, additional to that caused by the leakage proteolysis. Upon inhibition of transcription, the minimal threshold of Cyclin B1 needed to maintain the mitotic delay is more rapidly reached, resulting in the activation of the APC/C^Cdc20^ and the consequent active ubiquitination of Cyclin B1 and Securin, targeting these substrates for proteasome degradation. This hypothesis is further substantiated with our observation that inhibition of proteasome degradation does not prevent the decrease of Cyclin B1 mRNA levels upon inhibition of transcription, despite preventing the decrease of protein levels. Therefore, upon inhibition of proteasome degradation, inhibition of transcription or translation has no impact in the levels of Cyclin B1 protein pool, and accordingly, mitotic slippage no longer occurs.

Reinforcing this theory, we have shown that by increasing Cyclin B1 protein levels within the cells, fewer cells slip mitosis when transcription and translation are inhibited. One could expect a better rescue of the mitotic phenotype if the transfection efficiency was higher than 50%. However, we do not exclude the possibility that other protein(s) can play a role in the maintenance of the SAC and may also be regulated at the transcriptional and translational level during mitosis.

The requirement of protein synthesis during mitosis has already been proposed [15], [27], [28]. Recently, the continuous protein synthesis of Cdc20 in mitosis has been reported [28]. However, in our system the levels of Cdc20 mRNA do not change significantly when transcription is inhibited, excluding a role for Cdc20 in the induction of mitotic slippage. Although unclear, it is possible such discrepancy might result from different cell types used, namely human HeLa cells as opposed to murine NIH3T3 cells. To our knowledge, we present here the first experimental data that support the requirement of both transcription and translation for the sustained activation of the SAC, showing that at least *cyclin B1* gene expression is required. We propose that the maintenance of the SAC requires continuous transcription of mitotic driving regulators.

## Materials and Methods

### Cell culture, cell-cycle synchronization and cell treatments

NIH3T3 and HEK293 cells were cultured at 37°C, under 5% CO_2_, in DMEM (Dulbecco's modified Eagle's medium) with GlutaMAX™-I and 1000 mg/L glucose, supplemented with 10% (v/v) heat-inactivated FBS (foetal bovine serum) and 1% (v/v) penicillin-streptomycin (all reagents from Invitrogen). G1/S phases or G2/M phases-arrested cells were obtained after a 14 h treatment of an asynchronous population of cells with 2.5 mM thymidine (Sigma) or with 800 nM of nocodazole (Tocris), respectively. The treatments with actinomycin D (8 µM; Sigma), cycloheximide (35.5 µM; Sigma) and MG132 (25 µM; Tocris) were performed at 37°C for 4 h for NIH3T3 and for 6 h for HEK293 cells. All NIH3T3 and HEK293 cells were analyzed after 18 h and 20 h treatment, respectively. The drugs used were titrated and the minimal concentration of inhibition chosen.

### Immunofluorescence microscopy

NIH3T3 and HEK 293 cells were seeded on coverslips pre-treated with poly-L-lysine. Cells were washed with PBS and fixed with either 100% (v/v) methanol for 7 min at –20°C for subsequent immunofluorescence with α-Tubulin or with 4% (w/v) PFA (paraformaldehyde; Merck) for 20 min at room temperature for Cyclin B1 and Securin. After PBS washing from the fixative, cells were permeabilized with PBS-0.1% (v/v) Triton X100 for 3 min at room temperature, and blocked with PBS-3% (w/v) BSA for 30 min at room temperature. The primary antibodies were diluted in PBS-3% (w/v) BSA to a final concentration of 1∶200 (mouse anti-α-Tubulin, Sigma) or 1∶50 (mouse anti-Cyclin B1, Santa Cruz Biotechnology; mouse anti-Securin, Abcam) and incubated for 1 h at room temperature. Afterwards, cells were washed with PBS-0.01% (v/v) Tween 20 (Sigma) and incubated with anti-mouse-A488 antibody (Molecular Probes) diluted 1∶500 in PBS-3% (w/v) BSA. Cells were washed with PBS and counterstained with DAPI (4′,6-diamino-2-phenylindole dihydrochloride; 100 ng/ml; Sigma) for 5 min at room temperature. Coverslips were mounted on glass slides using Vectashield (Vector) mounting medium and sealed with nail-polish. Preparations were observed on a Leica DMRA2 fluorescence microscope with an objective of 1.4 numerical aperture and photographed with a Photometrics Cool Snap HQ Camera. Images were processed using Image J software (version 1.32a).

### Determination of mitotic and multinucleation index

DNA from NIH3T3 and HEK293 cells were stained with DAPI in a procedure similar to the described above. The mitotic index was measured by counting the number of cells with condensed chromosomes, and percentages calculated. Cells with more than one nucleus were counted as multinucleated cells. At least 4 fields comprising a total number of ≈100 cells were analyzed for each measurement. Slides were observed on a Leica DMRA2 fluorescence microscope.

### Protein extraction and Western blotting

All reagents except those specifically mentioned were purchased from Sigma. Cells were lysed in cold lysis buffer [25 mM HEPES, 5 mM MgCl_2_, 1 mM EGTA and 0.5% (v/v) Triton X-100, pH 7.5, supplemented with 2 mM NaF (sodium fluoride), 50 mM β-glycerol phosphate, 1 mM DTT (dithiothreitol), 2 mM PMSF (phenylmethanesulfonyl fluoride), 2 µg/ml aprotinin, 1.5 µg/ml benzamidine, 2 µg/ml leupeptin and 1 µg/ml pepstatin A] and centrifuged at 20000 x *g* for 15 min at 4°C. After protein quantification by the Bradford method using the Bio-Rad Protein Assay reagent (Bio-Rad), proteins were boiled at 100°C for 5 min with 6x SDS (sodium dodecyl sulphate) sample buffer [350 mM Tris/HCl, pH 6.8, 10% (w/v) SDS, 30% (v/v) glycerol, 0.6 M DTT, 0.12 mg/ml bromophenol blue]. Proteins were then analysed on 8–15% SDS-PAGE gels, and electroblotted onto nitrocellulose (Scheicher & Schuell) or PVDF (polyvinylidene fluoride; Bio-Rad) membranes. Membranes were blocked with PBS- 5% (w/v) non-fat dried milk for 1 h at room temperature and incubated for 1 h at room temperature or overnight at 4°C with mouse anti-Cyclin B1 (1∶200; [GNS1]; Santa Cruz Biotechnology), mouse anti-Cdc2/p34/CDK1 (1∶200; [17]; Santa Cruz Biotechnology), mouse anti-Securin (1∶200; [DCS-280]; Abcam), mouse anti-Bub3 (1∶1000; BD Transduction Laboratories), or mouse anti-β-Actin (1∶1000; Sigma) in blocking solution. Membranes were then repeatedly washed with PBS with 0.1% Tween 20 and incubated with anti-mouse-HRP antibody diluted 1∶1000 (Zymed Laboratories) for 1 h to overnight and washed with PBS. Detection of the signal was achieved using the ECL® (enhanced chemiluminescence) kit (Amersham Biosciences) according to the manufacturer's instructions. For antibody removal, membranes were incubated with stripping solution (62.5 mM Tris.HCl, pH 6.8, 0.5% (v/v) SDS, 0.875% (v/v) β-mercaptoethanol) for 30 min at 56°C and washed in PBS.

### Real-Time Quantitative PCR analysis

Total RNA was isolated from NIH3T3 using Trizol® reagent (Invitrogen™) and cDNA synthesis was performed using M-MuLV reverse transcriptase (Fermentas) according to manufacturer's instructions. cDNA from NIH3T3 cells was amplified using primers for mouse sequences of Bub3 (sense: 5′-CAGACTCGCTGCATCCGA-3′; antisense: 5′-CGGCCTTCGATGGAGCT-3′), BubR1 (sense: 5′-CGGGACGCAG GCCTC-3′; antisense: 5′-TGGGAAGCACGCTGGG-3′), Cdc20 (sense: 5′-CGATTT GCACTCACTGCTTCA-3′; antisense: 5′-CAGCGCGCAACCGG-3′), Cyclin B1 (sense: 5′-TGTGTGAACCAGAGGTGGAA-3′; antisense: 5′- CGGGCTTGGAGAGG GATTAT-3′), Mad1 (sense: 5′-ACTAGCCGTGGCCTCTGCT-3′; antisense: 5′-CAT CCCCAGTAGCTTGCTCC-3′), Mad2 (sense: 5′-GGTAGTGTTCTCCGTTCGATCT AGT-3′; antisense: 5′-GCAGGGTGATGCCTTGCT-3′), Securin (sense: 5′-TGAATG AAGAGAGAGGGCTGG-3′; antisense: 5′-AAAGGGTGTCTTCAGAGGGCTA-3′) and L19 (sense: 5′-GGAAAAAGAAGGTCTGGTTGGA-3′; antisense: 5′-TGATCTG CTGACGGGAGTTG-3′). *L19* expression served as internal control and was used to normalize for variances in input cDNA. Primers were designed using the ABI Primer Express software and their specificity confirmed using NCBI BLAST module. Detection of the expression of each gene was performed with SYBR Green (Applied Biosystems) in an ABI PRISM 7700 Sequence Detection System (Applied Biosystems), using the relative standard curve method. All measurements were performed in triplicate.

### Plasmids and transient transfections

YFP-CyclinB1-wt (kindly provided by Dr. R. Medema, Utrecht Medical Center, Utrecht, the Netherlands), GFP-CyclinB1-R42A (kindly provided by Dr. J Pines, The Wellcome Trust/Cancer Research UK Gurdon Institute, Cambridge, United Kingdom [29]), and pCMS-EGFP were used to transiently transfect HEK293 cells.

Twenty four hours before transfection, HEK293 cells were seeded at a density of 4×10^5^ cells per well in a 6 well-plate dishes, pre-treated with poly-L-lysine. Transfection of plasmid DNA (700 ng) was carried out using Lipofectamine 2000 (Invitrogen) according to the protocol provided with the product. Nineteen hours after the beginning of the transfection, cells were subjected to nocodazole treatment for 14 h. Further treatments with actinomycin D and cycloheximide were performed for 6 h at 37°C.

### Statistical analysis

Data are expressed as mean ± SD and were analysed for significance using Student's *t* test for comparison between two groups. P-values are as follows: *** p<0.005, ** p<0.01 and * p<0.05. All experiments were performed for at least three times.
